# Jinkui Shenqi Pill accelerates osteoporotic fracture healing by promoting bone formation through neurosensory PGE2/EP4/p-CREB axis

**DOI:** 10.3389/fendo.2025.1570685

**Published:** 2025-07-04

**Authors:** Jihao Xi, Danqing Fu, Dan Xu, Ruhan Shen, Yan Zhao, Haoqiang Dai, Chenjie Xia, Peihong Zhou

**Affiliations:** ^1^ Affiliated Cixi Hospital, Wenzhou Medical University, Ningbo, China; ^2^ Department of Orthopedic Surgery, The Affiliated Lihuili Hospital of Ningbo University, Ningbo, China; ^3^ School of Basic Medical Sciences, Zhejiang Chinese Medical University, Hangzhou, China; ^4^ Ningbo Municipal Hospital of Traditional Chinese Medicine, Affiliated Hospital to Zhejiang Chinese Medical University, Ningbo, China

**Keywords:** Jinkui Shenqi Pills, osteoporotic fracture, bone formation, sensory nerve, PGE2/EP4/p-CREB axis

## Abstract

**Purpose:**

Jinkui Shenqi Pill (JKSQP), a traditional Chinese herbal formula, is clinically utilized in China for managing bone disorders secondary to kidney deficiency, including osteoporotic fractures (OPFs). The present study aims to elucidate the pharmacological mechanism underlying JKSQP’s therapeutic effects on OPF healing.

**Methods:**

LC-MS/MS was employed to characterize the chemical constituents of JKSQP. Two-month-old female C57BL/6J mice underwent bilateral ovariectomy (OVX) followed by transverse tibial osteotomy to establish the OPF model. These OPF mice were randomly divided into the JKSQP group and OPF group, in which mice were gavaged with 1 g/kg/day JKSQP and equivalent volume of normal saline, respectively. At 4, 14, and 24 days post-fracture, biological specimens including serum, tibiae, dorsal root ganglion (DRG) and hypothalamus were collected for ELISA assay, μCT analysis and histopathology staining. Primary bone marrow stromal cells (BMSCs) were treated with the serum obtained from Sprague-Dawley rats administered with 1.5 g/kg/day JKSQP via oral gavage for three consecutive days. The conditioned medium derived from these JKSQP serum-treated BMSCs and the serum collected from the JKSQP-treated mice were applied to the DRG neurons. The levels of COX-2, PGE2, EP4 and CGRP *in vitro* were detected using qRT-PCR, western blot, ELISA and immunofluorescence (IF).

**Results:**

LC-MS/MS analysis identified 1872 chemical components in JKSQP. μCT evaluation demonstrated accelerated healing of OPF in JKSQP-treated mice. Histomorphometric analysis combined with Calcein double-labeling revealed enhanced bone formation within the fracture callus. Compared with OPF controls, mice in the JKSQP group exhibited elevated serum PGE2 levels, upregulated Osterix, COX-2 and EP4 expression in fracture callus, increased EP4 and CGRP in DRG, and enhanced p-CREB in hypothalamus. *In vitro*, JKSQP-containing serum increased both PGE2 secretion and COX-2 expression in BMSCs. Furthermore, qRT-PCR and IF analyses confirmed that both conditioned medium from JKSQP-treated BMSCs and serum from JKSQP-administered mice upregulated EP4 and CGRP expressions in DRG neurons.

**Conclusion:**

Jinkui Shenqi Pill accelerates OPF healing by promoting bone formation possibly through activation of neurosensory PGE2/EP4/p-CREB axis.

## 1 Introduction

Osteoporosis is a common skeletal disease characterized by decreased bone mass and deterioration of bone structure, resulting in an increased risk of fragility fracture (1). Osteoporotic fractures (OPFs) frequently occurring in the spine, hip and distal radius, affect more than 9 million individuals worldwide, particularly among post-menopausal women. OPFs are associated with a high risk of disability and mortality because osteoporotic bones heal poorly and tend to break into complex fracture patterns (2). The management of OPFs costs billions of dollars annually, placing a substantial burden on both national economies and healthcare systems (3). Current drugs for OPFs have limited clinical utility due to dose-dependent adverse effects such as osteonecrosis and gastrointestinal complications (4). In contrast, natural productions with relative efficacy and safety are emerging as promising candidates for developing novel OPF therapeutics (5).

OPF healing is a complex process involving a tight coordination of diverse cell types (6). Following an initial inflammatory immune response at the fracture site, bone marrow stromal cells (BMSCs), a heterogeneous population comprising mesenchymal stem cells, macrophages, endothelial cells and other, are recruited (7). Within the fracture gap, BMSC-derived stem cells differentiate into chondrocytes to form a temporary cartilaginous template. This template undergoes chondrocyte apoptosis, chondrocyte-to-osteoblast transdifferentiation, matrix calcification and bone-forming osteoblasts invasion, and is finally replaced by bone (8). Concurrently, at the rims of the fracture callus, BMSCs directly differentiate into osteoblasts to form woven bone (9). Bone formation is a crucial step for successful fracture healing, as bony bridging not only restores the mechanical properties of broken bone but also enable subsequent osteoclast-mediated remodeling (10).

Bone is richly innervated by sensory nerves which is essential for bone regeneration (11). Massive evidence showed that sensory denervation leads to a mechanically insufficient fracture callus because of poor osteogenesis (12). Following OPF, various inflammatory mediators are largely produced, among which prostaglandin E2 (PGE2) exerts bone anabolic effects by sensory nerves (13). The synthesis of PGE2 is controlled by the limiting enzyme cyclooxygenase 2 (COX-2). After PGE2 binds to EP4 receptor on sensory nerves, the signal transmits through dorsal root ganglion (DRG) to phosphorylate cAMP response element binding (CREB) in hypothalamus for osteogenesis (13, 14). Thus, this neurosensory PGE2/EP4/p-CREB axis is regarded as a potential therapeutic target to accelerate OPF healing.

Jinkui Shenqi Pill (JKSQP) first recorded in *Synopsis of Golden Chamber* (金匮要略), is a classical prescription composed of 8 herbs (Listed in **Table 1**). JKSQP has effects of tonifying kidney, and thus has been widely used to treat various kidney deficiency-induced diseases for thousands of years (15). OPFs are also called fragility fracture as they are caused even under a slight trauma. According to the Chinese medicine theory of “kidney governing bones” (16), the pathologic changes including bone loss and deterioration in OPFs are thought to be rooted in kidney deficiency (5, 17). Clinical application of JKSQP has obtained satisfactory outcomes on acceleration of OPF healing (18), while its pharmacological mechanism remains largely unclear. Here, we hypothesized that JKSQP accelerates OPF healing by promoting bone formation via neurosensory PGE2/EP4/p-CREB axis, and animal and cellular experiments were performed to test this hypothesis.

**Table 1:** The compositions of JKSQP.

Chinese name	Latin Name	Parts used	Dosage, g
Dihuang	*Rehmannia glutinosa*	Root	24
Shanyao	*Dioscorea batatas*	Root	12
Shanzhuyu	*Cornus officinalis*	Fruit	12
Fuling	*Wolfiporia cocos*	Sclerotium	9
Mudanpi	*Paeonia moutan*	Bark	9
Zexie	*Rhizoma Alismatis*	Rhizome	9
Guizhi	*Cinnamomum cassia*	Bark	3
Fuzi	*Aconitum carmichaelii*	Root	3

## 2 Materials and methods

### 2.1 LC-MS/MS analysis and administration of JKSQP

JKSQP is composed of 24g *Rehmannia glutinosa* (Dihuang), 12g *Rehmannia glutinosa* (Shanyao), 12g *Cornus officinalis* (Shanzhuyu), 9g *Wolfiporia cocos* (Fuling), 9g *Paeonia moutan* (Mudanpi), 9g *Rhizoma alismatis* (Zexie), 3g *Cinnamomum cassia* (Guizhi) and 3g *Aconitum carmichaelii* (Fuzi). JKSQP in the form of Chinese patent medicine was purchased from Beijing Tongrentang Co., Ltd, and made into the solution by mixed with pure water. Liquid chromatography-tandem mass spectrometry (LC-MS/MS) was performed to analyze the chemical ingredients in the solution of JKSQP. According to the dose conversion factor between human beings and mice, JKSQP was orally gavaged at the dosage of 1 g/kg/day.

### 2.2 Mice and grouping

A total of 78 female C57BL/6J mice (2-month-old, weighing 22 ± 2g) were purchased from the Experimental Animal Center of Zhejiang Chinese Medical University. The mice were housed in a barrier facility under controlled conditions: a 12-hour light/dark cycle, constant temperature of 25°C and 40-60% humidity, and had free access to water and food. After 1 week of adaptive feeding, the mice were divided into 4 groups including the sham group (n=12), the OVX group (n=8), the OPF group (n=29), and the JKSQP group (n=29). Mice in the OVX group underwent bilateral ovariectomy (OVX), whereas the sham group received surgical removal of an equivalent volume of periovarian adipose tissue. Femoral samples were collected from sham-operated and OVX mice at 8 weeks post-surgery for micro-computed tomography (μCT) analysis. The remaining two groups of mice underwent bilateral OVX followed by tibial osteotomy 8 weeks post-initial surgery to establish OPF model. Mice in the JKSQP group received daily oral gavages of JKSQP (1 g/kg/day, converted from human adult doses via body surface area), while the OPF group received equal-volume saline. The mice in both the JKSQP and OPF groups were sacrificed at 4, 14 and 24 days post-fracture (n=8 per group per time point), and their biological specimens including serum, tibiae, DRG, and hypothalamic tissues were collected for subsequent analyses. The 5 mice remaining in per OPF and JKSQP groups were sacrificed at 14 days post-fracture for Calcein double-labeling. No mice died accidentally or for other factors in the process of experiment.

### 2.3 OPF model

Mouse OPF model was established as previous described (19, 20). C57BL/6J mice were anesthetized by continuous inhalation with isoflurane, and bilateral ovariectomy (OVX) was performed via two small incisions on either side of the lower back. After 8 weeks of OVX surgery, the femurs were scanned by micro-computed tomography (μCT) to detect bone loss in mice. Then, these OVX mice underwent a unilateral transverse tibial fracture. Briefly, an incision of 1 cm was made along the surface of tibial crest. Through the tibial platform, a 26-gauge needle was longitudinally inserted into tibial cavity. After removal of the needle, tibia was transversely cut by a NO.11 surgical blade at the midpoint. Then, the transverse fracture was fixed again by the needle.

### 2.4 μCT analysis

Femur and fractured tibia tissues were scanned with μCT (Skyscan1176, Belgium) at a resolution of 9 μm. Image reconstruction was performed using NRecon software with parameters set to 6 for ring artifact correction and 30% for beam hardening correction, followed by three-dimensional (3D) reconstruction in CTVol software. For femoral morphometric analysis, a region of interest (ROI) was defined in the distal femur metaphysis with cortical bone excluded. Quantitative parameters including bone mineral density (BMD, g/cm³), bone volume fraction (BV/TV, %), and trabecular separation (Tb.Sp, mm) were analyzed to assess osteoporotic changes. For fracture healing evaluation, the medial side fracture callus excluded cortical bone was selected as a ROI. Total bone volume (mm³) and BV/TV were calculated as previously described (8).

### 2.5 Histology and histomorphometry

After fixation with 4% paraformaldehyde (Beijing Solebio Technology Co., LTD., China), dehydration with gradient alcohol and decalcification with 14% EDTA solution, tibia samples were processed for paraffin sections of 3-μm-thick as previously described (21). The sections were stained with Alcian Blue Hematoxylin (ABH)/Orange G for morphological analysis. By using OsteoMetrics software (Decatur, GA, USA), the percentage of cartilage callus area over the total fracture callus area (Cg.Ar/Ps.CI.Ar, %) and the percentage of woven bone area over the total fracture callus area (Md.Ar/Ps.CI.Ar, %) were calculated to evaluate enchondral bone formation during OPF healing.

### 2.6 Calcein double-labeling

Mice in each of the OPF and JKSQP groups were intraperitoneally injected with 10 mg/kg calcein (Sigma, St. Louis, USA) 7 days and 12 days post-fracture, and sacrificed at 14 days post-fracture. After fixation and dehydration, tibia samples were embedded with polymethyl methacrylate, and cut into 20 μm sections for fluorescence examination. Mineral apposition rate (MAR) and bone formation rate/bone surface (BF/BS) were measured using Image J software.

### 2.7 Preparation of JKSQP-containing serum and BMSCs intervention

Six 2-month-old Sprague-Dawley (SD) rats were obtained from the Experimental Animal Center of Zhejiang Chinese Medical University and administered 1.5 g/kg/day JKSQP or normal saline via oral gavage for three consecutive days. Whole blood was collected through abdominal aorta puncture under anesthesia and centrifuged at 200 ×g for 10 minutes at 4°C to separate serum. The JKSQP-containing serum and JKSQP-free serum from saline-treated rats were stored at -80°C for subsequent cellular experiments.

BMSCs were isolated from the proximal femur of 2-month-old C57BL/6J mice. After centrifugation at 200 ×g for 10 minutes, cells were resuspended with α-MEM (Gibco, Grand Island, NY) containing 10% fetal bovine serum (FBS, Gibco). Through changing the medium 48 hours later, non-adherent cells were removed and the primary BMSCs were harvested. Next, BMSCs were treated with JKSQP-containing serum (250 μL and 750 μL doses), using JKSQP-free serum as the control. Following a 24-hour intervention, both BMSCs and culture medium were collected for western blot analysis and ELISA, respectively.

### 2.8 Enzyme-linked immunosorbent assay analysis

Mouse blood samples from the OPF group and JKSQP group were obtained by eyeball extirpating, and centrifuged at 200 × g for 30 min to extract serum. The conditional culture medium were collected after the intervention of BMSCs. The concentration of PGE2 in both mouse serum and conditioned medium were subsequently measured according to the instructions of the serum Elisa kit (Jiancheng Bioengineering Institute, Nanjing, China).

### 2.9 Western blot analysis on BMSCs

BMSCs from each group were lysed by protein extraction reagent. Total protein were obtained and quantified by a BCA Protein Assay Kit (Beyotime, CN). Next, protein samples were separated by electrophoresis and transferred to PVDF membranes (BIO-RAD, USA). After blocking for 20 min, the membranes were incubated overnight at 4°C with primary antibody of COX-2 (diluted 1:1000, Abcam, ab15191, UK). Following washing with TBST, the membranes were incubated with secondary antibodies for 2 hour. The immune complexes were detected with a chemiluminescent HRP substrate and visualized via the Chemiluminescence Image Analysis System (Tanon 5200, CN).

### 2.10 Mouse DRG neuron isolation, identification and intervention

The vertebral column was isolated from 8-week-old C57BL/6 mice anesthetized by an overdose of isoflurane. Bilateral DRGs in lumbar 1–3 were localized and collected from intervertebral foramina under a stereomicroscope. The DRGs were digested with the enzymatic solution containing 5 mg/mL collagenase type I (GIBCO, 17100-017, USA) and 5 mg/mL dispase type II (Yuanye Biotechnology, S25046, CN) for 1 hour in a water bath at 37°C. The resulting cell suspension was filtered by a 200 µm cell strainer to remove undigested tissue debris. After centrifugation at 200 ×g for 10 minutes, the enzymatic solution was aspirated, and the cell suspension was resuspended in α-MEM medium (Gibco, Grand Island, NY) containing 10% FBS (Gibco) and 1 μM 5-fluoro-2’-deoxyuridinem, and then plated on fibronectin-coated petri dishes for cell adherence.

After 5–7 days of cultivation, glial proliferation could be inhibited by 5-fluoro-2’-deoxyuridine, yielding purified DRG neurons for subsequent identification and intervention. Two biomarkers, β3-tubulin stained neuronal cytoskeleton and glial fibrillary acidic protein (GFAP) used to exclude astrocytes, were employed to identify DRG neurons via IF staining. The DRG neurons were treated for 24 hours with serum obtained from JKSQP-treated mice or OPF controls, and conditioned medium from BMSCs exposed to either JKSQP-containing serum or control serum.

### 2.11 Quantitative gene expression analysis on DRG neurons

Quantitative Reverse Transcription Polymerase Chain Reaction (qRT-PCR) were performed on DRG neurons as previous described (22). Total RNA was isolated from DRG neurons using RNA-Quick Purification Kit (TransGen Biotech, Beijing, CN) according to the manufacturer’s recommendations. After quantification with the photometric method, 500 ng of total RNA was reverse-transcribed into cDNA using PrimeScript™ RT reagent Kit (Takara, Japan). Real-time PCR was performed using Green^®^ Premix Ex Taq™ II FAST (Takara, Japan) and the gene primer sequences (List in **Table 2**) on a QuantStudio™ 5 real-time PCR system (Applied Biosystems, USA). Gene expression analysis was conducted using the 2−ΔΔCt method, and GAPDH was used as the internal control.

**Table 2:** The primer name and sequences for PCR analysis.

Primer name	Forward	Reverse
GAPDH	GGTTGTCTCCTGCGACTTCA	TGGTCCAGGGTTTCTTACTCC
EP1	GGGCTGGAACTCTAACTCCC	GTGACTGAAACCACTGTGCC
EP2	GGAATTGGTGCTCACTGACC	GCATCGTGGCCAGACTAAAG
EP3	GGTCATCCTCGTGTACCTGT	CTTCATGTGGCTGGCATACC
EP4	GCCCGGGAGTTAAAGGAGAT	TCCACCAACAGGACACTCTC

### 2.12 Immunohistochemistry and immunofluorescence assay

The tibia sections were treated with 0.01 mol/L citrate buffer at 60°C for 4 hours as antigen retrieval. Then, the sections were incubated in the primary antibodies of osterix (diluted 1:300, Abcam, ab22552, UK), COX-2 (diluted 1:200, Abcam, ab15191, UK), EP4 (diluted 1:200, Abcam, ab92763, UK), Calcitonin gene-related peptide (CGRP, diluted 1:200, Abcam, ab81887, UK), p-CREB (diluted 1:200, Abcam, ab32096, UK) overnight at 4°C. After reaction with secondary antibodies including Goat anti-mouse/rabbit IgG-HRP (diluted 1:1000, Beijing Zhongshan Jinqiao Biotechnology, Beijing, China), Goat anti-mouse/rabbit IgG H&L (Alexa Fluor^®^ 488) (diluted 1:500, Abcam, UK) for 20 minutes, the sections were stained with diaminobenzidine solution followed by hematoxylin for IHC assay, or stained with 4’,6-diamidino-2-phenylindole (DAPI) for IF assay. The quantification of positive area was calculated using Image-Pro Plus software (Media Cybernetics, Silver Spring, USA).

Primary DRG neurons were cultured on poly-L-lysine-coated coverslips. After fixation with 4% paraformaldehyde and blocking with 3% bovine serum albumin for each 30 minutes, the coverslips were incubated with primary antibodies including β3-tubulin (diluted 1:300, wanleibio, wl03860, CN), GFAP (diluted 1:100, wanleibio, wl0836, CN), EP4 (diluted 1:200, abcam, ab92763, UK) and CGRP (diluted 1:200, Abcam, ab81887, UK) overnight at 4°C. Then, a secondary antibody (Alexa Fluor^®^ 488, abcam, UK) and DAPI were applied on the coverslips for IF staining. Quantitative analysis of positive staining in mouse tissues and DRG cells were evaluated using Image-Pro Plus software (Media Cybernetics, Silver Spring, USA).

### 2.13 Statistical analysis

All data were presented as mean ± standard deviation. One-way ANOVA followed by Dunnett’s test was performed using SPSS 20.0 software. The value of p < 0.05 was considered statistically significant. *p < 0.05, **p < 0.01 and ***p < 0.001; “ns” indicated non-significance.

## 3 Results

### 3.1 Chemical component analysis of JKSQP by LC-MS/MS

**Figure 1:**
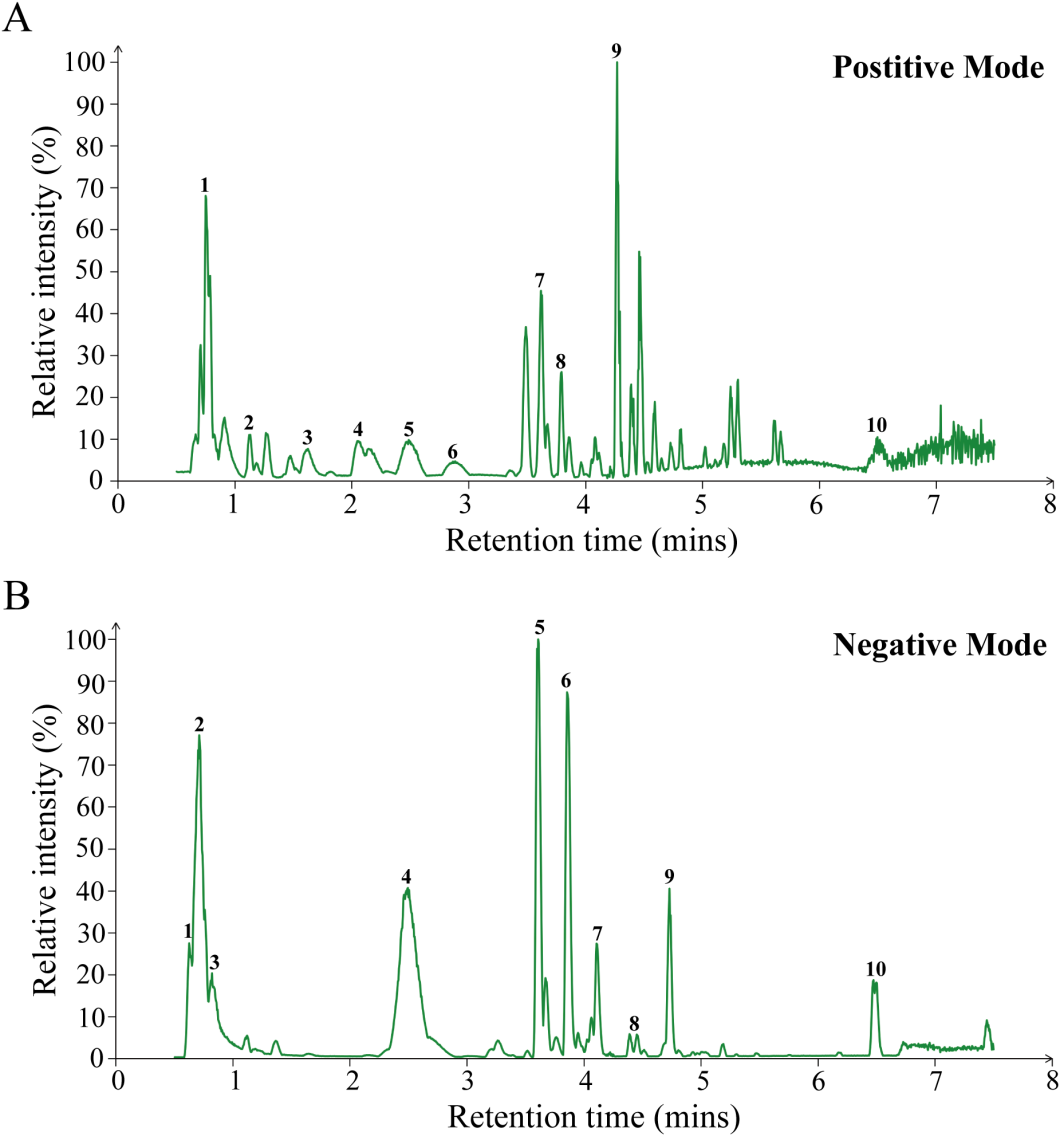
LC-MS/MS analysis of JKSQP compounds. **(A)** Base peak chromatogram in positive ion mode. Peak assignments: 1. Arginine; 2. L-glutamine; 3. Melamine; 4. Karacoline; 5. D-glucuronate; 6. Zongorine; 7. Phlorisobutyrophenone; 8. Talatizamine; 9. Benzoylmesaconine; 10. Octadecanamide;. **(B)** Base peak chromatogram in negative ion mode. Peak assignments: 1. Cis-aconitic acid; 2. L-malic acid; 3. Quinic acid; 4. Lotaustralin; 5. Geniposide; 6. Verbascoside; 7. Cornuside; 8. Benzoyloxypeoniflorin; 9. Trans-cinnamic acid; 10. Hyperforin;.

The chemical components in the aqueous solution of JKSQP were qualitatively analyzed using LC-MS/MS. A total of 1872 chemical components were identified, with 1185 components detected in positive ionization mode (**Figure 1A**) and 687 components in negative ionization mode (**Figure 1B**). Twenty representative compounds from both ionization modes are listed in **Table 3**.

**Table 3:** The detailed analyte parameters of LC-MS/MS.

Compound name	Pos/Neg	m/z	RT(s)	Exact mass	Formula	CAS
Arginine	Pos	175.1196	42.9	174.11167	C6H14N4O2	74-79-3
L-glutamine	Pos	130.05	67.7	146.06914	C5H10N2O3	56-85-9
Melamine	Pos	127.0395	97.4	126.06539	C3H6N6	1246816-14-7
Karacoline	Pos	378.2652	130.5	377.2566	C22H35NO4	39089-30-0
D-glucuronate	Pos	195.0659	149.8	194.04265	C6H10O7	6556-12-3
Zongorine	Pos	358.2383	174	357.23038	C22H31NO3	509-24-0
Phlorisobutyrophenone	Pos	197.0818	220.4	196.0735552	C10H12O4	35458-21-0
Talatizamine	Pos	422.2887	227.7	421.28281	C24H39NO5	20501-56-8
Benzoylmesaconine	Pos	590.2899	256.7	589.28868	C31H43NO10	63238-67-5
Octadecanamide	Pos	284.2964	391	283.2875	C18H37NO	124-26-5
Cis-aconitic acid	Neg	173.0091	41.7	174.01644	C6H6O6	585-84-2
L-malic acid	Neg	133.0147	43.1	134.02152	C4H6O5	97-67-6
Quinic acid	Neg	191.0548	46.4	192.06339	C7H12O6	36413-60-2
Lotaustralin	Neg	243.0884	151.5	261.1212314	C11H19NO6	1973415-50-7
Geniposide	Neg	433.1385	218.9	388.13694	C17H24O10	24512-63-8
Verbascoside	Neg	623.1981	237.2	624.20541	C29H36O15	61276-17-3
Cornuside	Neg	541.1573	247.1	542.16355	C24H30O14	131189-57-6
Benzoyloxypeoniflorin	Neg	599.1789	266.4	600.18428	C30H32O13	72896-40-3
Trans-cinnamic acid	Neg	193.0517	279.5	148.05243	C9H8O2	140-10-3
Hyperforin	Neg	535.3789	389.6	536.38654	C35H52O4	11079-53-1

Pos, positive ion mode; Neg, negative ion mode; m/z, mass-to-charge ratio; RT, Retention time.

### 3.2 JKSQP accelerates OPF healing in mice

**Figure 2:**
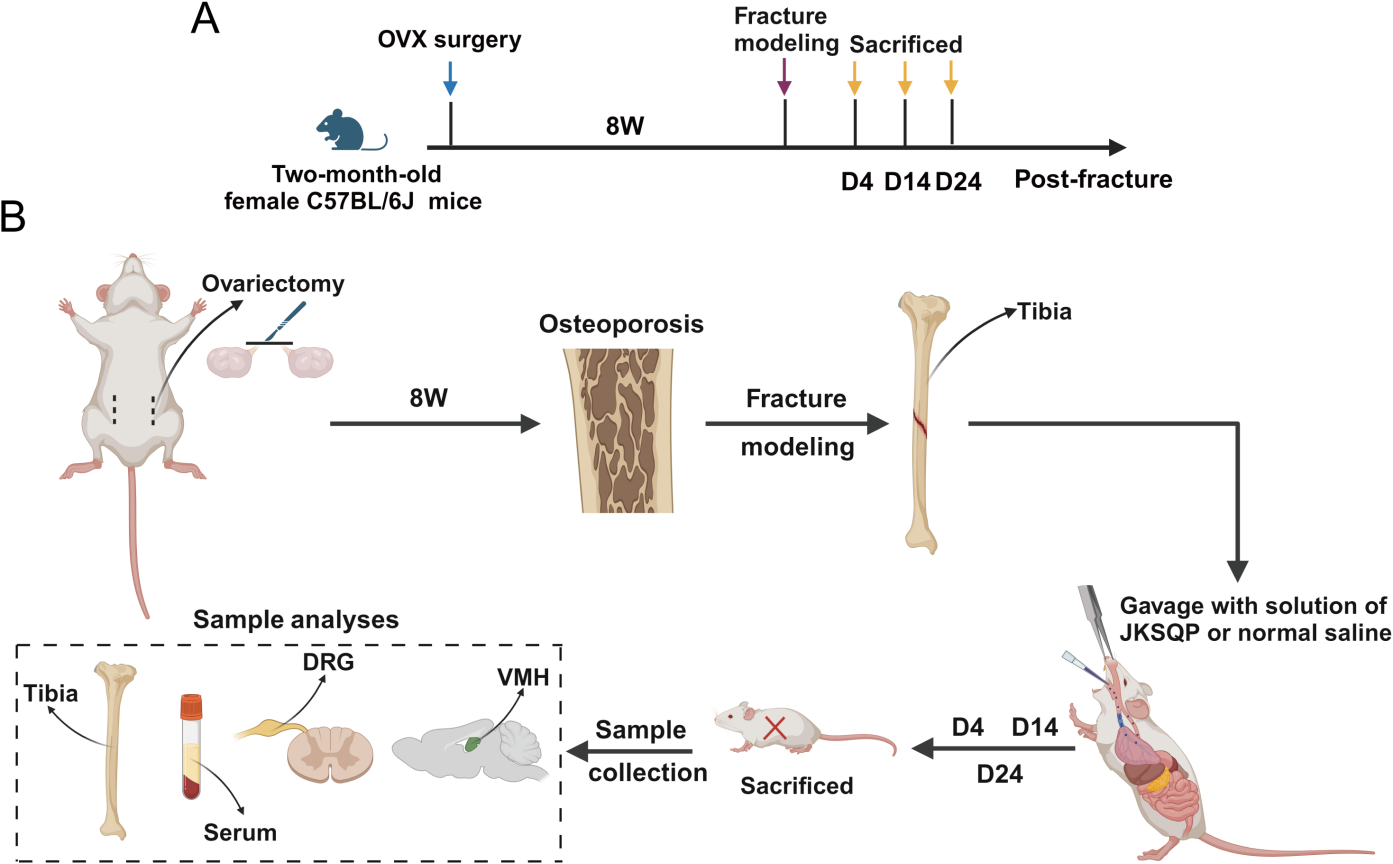
Experimental timeline of the OPF model. **(A)** Two-month-old female C57BL/6J mice received underwent bilateral ovariectomy (OVX) followed by tibial osteotomy 8 weeks post-initial surgery to establish OPF model. **(B)** Samples including serum, fractured tibiae, dorsal root ganglion (DRG) and hypothalamic tissue were obtained at 4, 14 and 24 days post-fracture for subsequent analyses.

To investigate the pharmacological mechanism of JKSQP on OPF healing, *in vivo* animal experiments were conducted following the experimental timeline outlined in **Figure 2**. Two-month-old female C57BL/6J mice underwent bilateral OVX followed by tibial osteotomy to establish an OPF model. Biological samples including serum, fractured tibiae, DRGs, and hypothalamic tissues, were collected at postoperative days 4, 14, and 24 for subsequent biochemical and histological analyses.

**Figure 3:**
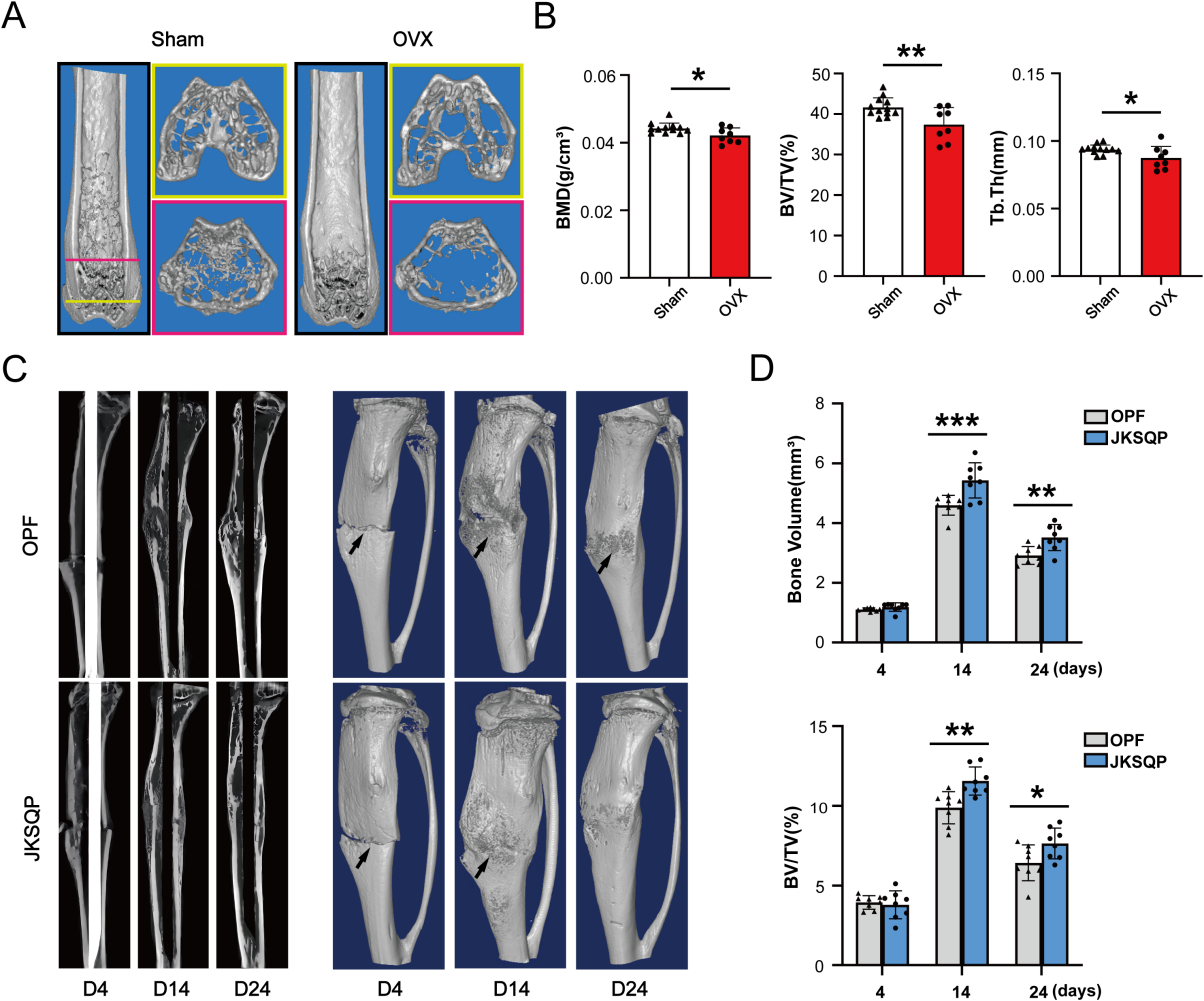
JKSQP accelerates OPF healing in mice. **(A)** Three-dimensional reconstruction images of μCT showed severe bone loss in the OVX mice compared to sham-operated controls at 8 weeks post-surgery. **(B)** Quantitative analyses of BMD, BV/TV and Tb.Th in the distal femur metaphysis. **(C)** μCT images showed fracture callus formation in the JKSQP treated mice at 4, 14 and 24 days post-fracture. The black arrowhead indicates the fracture gap. **(D)** Quantification of callus bone volume and BV/TV in JKSQP-treated versus vehicle control groups. *p < 0.05, **p < 0.01, ***p < 0.001.

Initial validation of osteoporosis induction was performed by harvesting femurs 8 weeks post-ovariectomy (OVX). μCT analysis revealed significant bone loss in OVX mice (**Figure 3A**), with quantitative metrics of reduced BMD, BV/TV and Tb.Th (**Figure 3B**). Subsequently, tibial osteotomy was performed on the osteoporotic mice to establish the OPF model. μCT images showed a blurry fracture gap at 14 days post-fracture in the JKSQP-treated mice compared to saline-treated OPF controls (**Figure 3C**, **Supplementary Figure 1**). Complete fracture union was achieved by day 24 in the JKSQP group, whereas persistent gaps remained in untreated OPF mice (**Figure 3C**, **Supplementary Figure 1**). Quantitative morphometry showed increased callus bone volume and BV/TV in JKSQP-treated mice during healing (**Figure 3D**). These data demonstrate JKSQP’s therapeutic efficacy in accelerating OPF healing.

### 3.3 JKSQP promotes bone formation in the fracture callus of OPF mice

**Figure 4:**
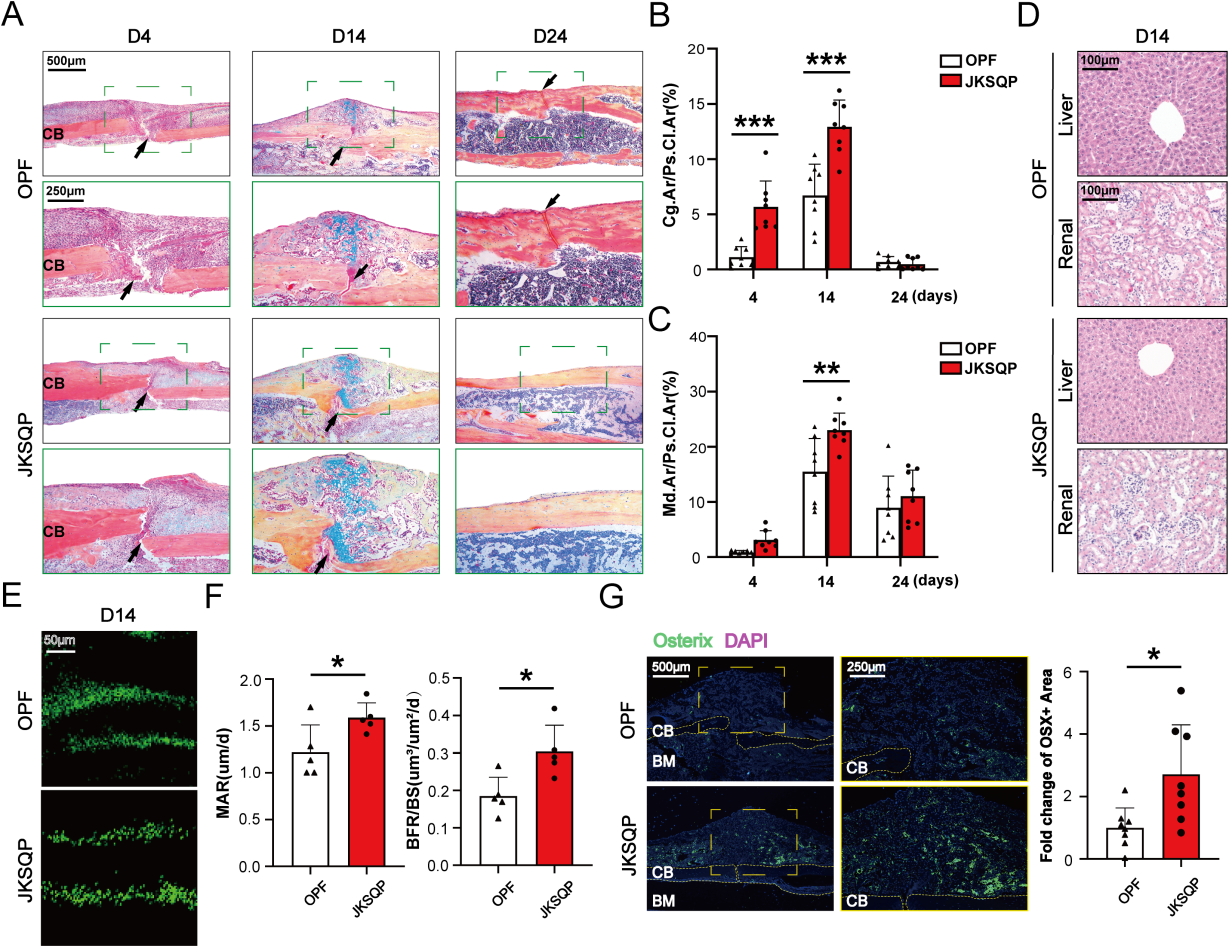
JKSQP promotes bone formation in OPF mice. **(A)** Alcian blue/hematoxylin (ABH) staining showed a large fracture callus at 14 days post-fracture and complete bridging of the fracture gap at 24 days post-fracture in the JKSQP-treated mice. The black arrowhead indicates the fracture gap. **(B)** Quantification of cartilage-to-callus area ratio (Cg.Ar/Ps.Cl.Ar, %). **(C)** Quantification of woven bone-to-callus area ratio (Md.Ar/Ps.CI.Ar, %). **(D)** Hematoxylin and eosin (H&E) staining of kidney and liver tissues from JKSQP-treated mice and OPF controls. **(E)** Calcein double-labeling showed enhanced mineralized tissue formation in the JKSQP-treated callus at 14 days post-fracture. **(F)** Quantitative analysis of mineral apposition rate (MAR, μm/day) and bone formation rate/bone surface (BF/BS, μm³/μm²/day). **(G)** Immunofluorescence (IF) staining of Osterix in fracture callus with corresponding quantitative analysis. *p < 0.05, **p < 0.01, ***p < 0.001.

Successful fracture healing requires coordinated bone formation (23). To evaluate JKSQP’s osteogenic effects, tibial tissues from OPF mice were subjected to histopathological analysis. ABH/Orange G staining and quantitative analyses demonstrated enlarged cartilage callus at 4 and 14 days post-fracture, followed by increased mineralized callus at 14 days in JKSQP-treated mice (**Figures 4A–C**). At 24 days post-fracture, complete fracture union was observed in the JKSQP group, whereas residual callus persisted in untreated OPF controls (**Figures 4A–C**). Hematoxylin and eosin (H&E) staining of kidney and liver tissues revealed no significant structural alterations in JKSQP-treated mice compared to OPF controls, suggesting an absence of toxicity or adverse effects associated with JKSQP administration in mice (**Figure 4D**). Calcein double-labeling and quantitative analysis of MAR and BF/BS revealed accelerated bone-formation rates in JKSQP-treated callus at 14 days (**Figures 4E, F**). Osterix is an osteoblast-specific transcription factor, essential for osteoblast differentiation and bone formation. IF staining showed upregulation of Osterix in the JKSQP-treated mice (**Figure 4G**). These findings demonstrate that JKSQP promotes OPF healing by enhancing bone formation.

### 3.4 JKSQP activates COX-2/PGE2 axis in the fracture callus during OPF healing

**Figure 5:**
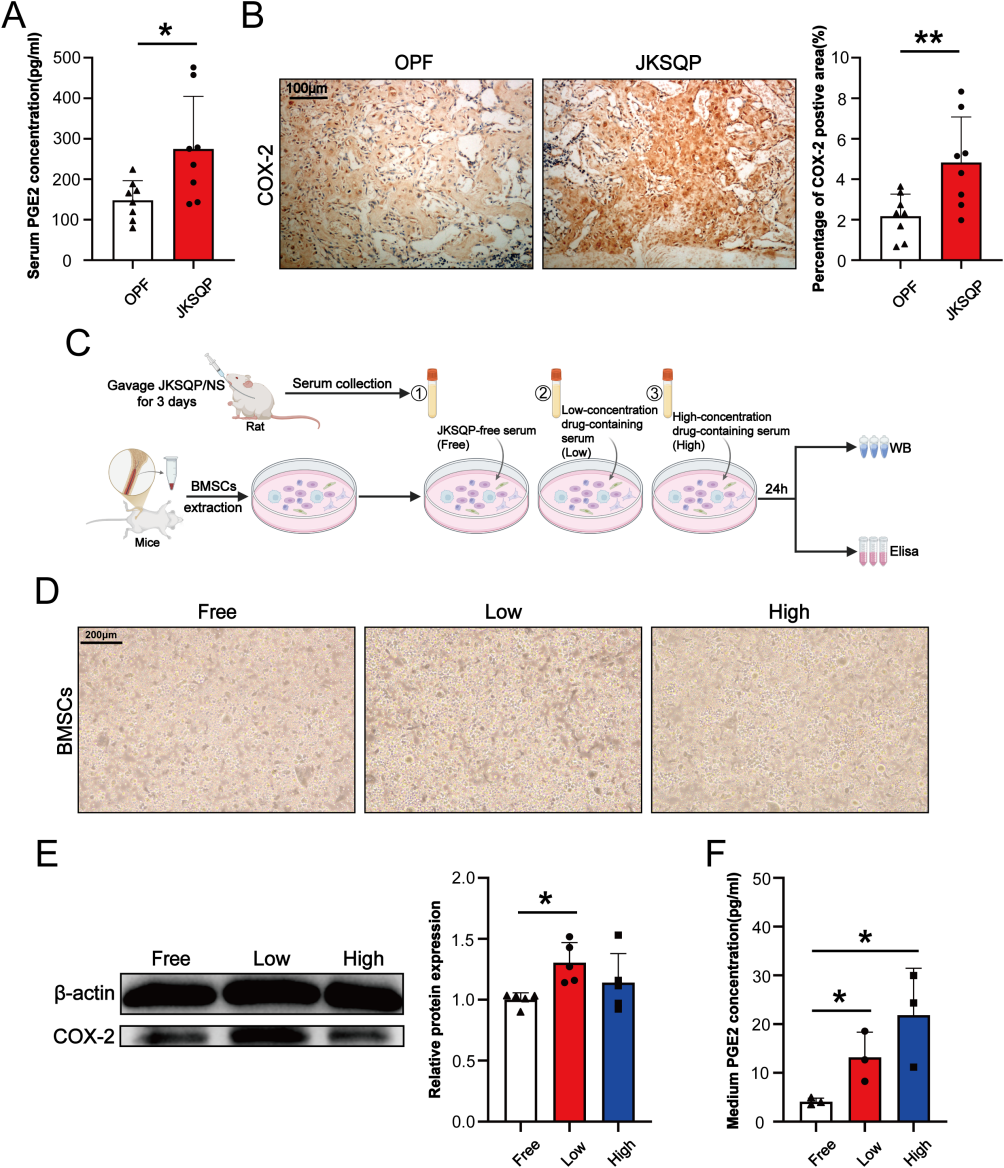
JKSQP upregulates COX-2/PGE2 signaling during OPF healing. **(A)** Serum PGE2 levels in JKSQP-treated versus control mice at 14 days post-fracture. **(B)** Immunohistochemical staining of COX-2 in fracture callus and quantification of COX-2-positive area. **(C)** The flowchart of primary bone marrow stromal cells (BMSCs) extraction and intervention with JKSQP-free serum, low and high concentration of JKSQP-containing serum. **(D)** Phase-contrast microscopy of primary BMSCs in each group. **(E)** Western blot analysis of COX-2 expression in BMSCs post-intervention. **(F)** PGE2 concentration in conditioned medium from BMSCs post-intervention. *p < 0.05, **p < 0.01.

Multiple lines of evidence have demonstrated the importance of neurosensory PGE2/EP4/p-CREB axis in regulating bone formation (13, 14). To determine the pharmacological mechanism underlying JKSQP’s effect on OPF, we firstly detected the expressions of COX-2 and PGE2 in the OPF mice. COX-2 is the rate-limiting enzyme controlling PGE2 synthesis. The results of ELISA assay and IHC staining showed that serum PGE2 and COX-2 positive area in the fracture callus were significantly increased in the JKSQP treated mice (**Figures 5A, B**). It is well known that BMSCs are composed of heterogeneous cell populations including mesenchymal stem cells, osteoblasts, macrophages, endothelial cells, and others, and serve as a critical source of PGE2 synthesis within bone (24). To determine whether JKSQP promotes PGE2 synthesis in BMSCs, primary BMSCs were cultured and then treated for 24 hours with JKSQP-free rat serum, low or high concentration of JKSQP-containing rat serum (**Figure 5C**). No significant morphological differences were observed between the JKSQP-free group and low- or high-concentration JKSQP group (**Figure 5D**), indicating absence of JKSQP cytotoxicity in BMSCs. Western blot analysis demonstrated significantly upregulated COX-2 expression in BMSCs treated with JKSQP-containing serum (**Figure 5E**). ELISA quantification revealed elevated PGE2 levels in the culture medium following treatment with JKSQP-containing serum (**Figure 5F**). Collectively, these findings indicate that JKSQP activates the COX-2/PGE2 axis pathway within the fracture callus during OPF healing.

### 3.5 JKSQP enhances EP4 expression in DRG and p-CREB levels in hypothalamus in OPF mice

**Figure 6:**
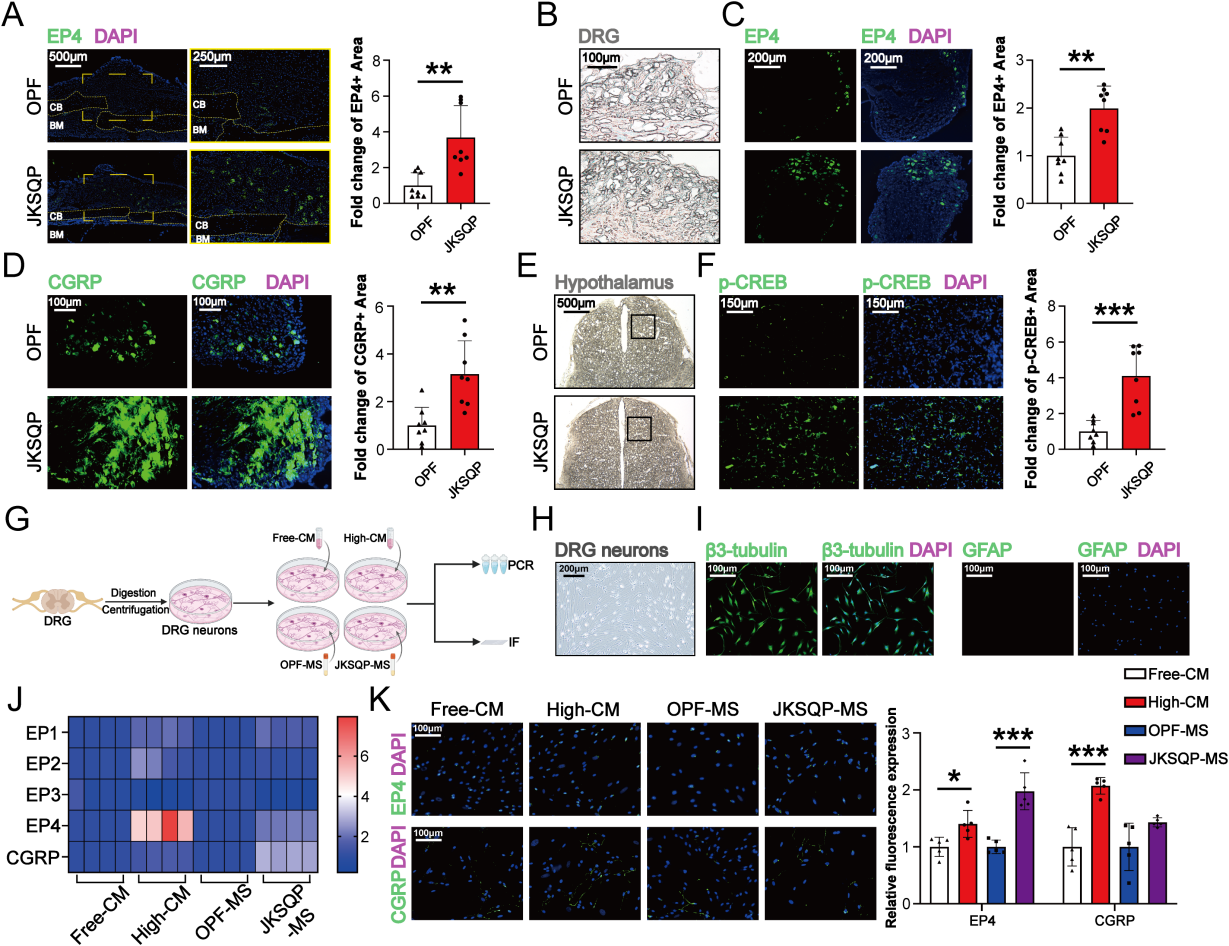
JKSQP promotes EP4 in DRG and p-CREB in hypothalamus during OPF healing. **(A)** Immunofluorescence (IF) staining of EP4 in the fractured callus of OPF mice at the 14 days post-fracture. **(B)** Histomorphology of mouse dorsal root ganglion (DRG) at 14 days post-fracture. **(C, D)** IF staining and quantitative analysis of EP4 and CGRP in DRG at 14 days post-fracture. **(E)** Histomorphology of mouse brain at 14 days post-fracture. Black boxes: hypothalamus region. **(F)** IF staining and quantitative analysis of p-CREB in hypothalamus. **(G)** The flowchart of primary dorsal root ganglion (DRG) neurons cultivation and intervention. Free-CM: JKSQP-free serum treated BMSCs-conditioned medium; High-CM: high JKSQP-containing serum treated BMSCs-conditioned medium; OPF-MS: the serum from OPF mice; JKSQP-MS: the serum from JKSQP-treated mice. **(H)** Phase-contrast microscopy of DRG neurons. **(I)** IF staining of β3-tubulin and glial fibrillary acidic protein (GFAP) in DRG neurons. **(J)** Heat map of EP1-EP4 mRNA levels in DRG neurons post-intervention. **(K)** IF staining of EP4 and CGRP in DRG neurons post-intervention. *p < 0.05, **p < 0.01, ***p < 0.001.

After PGE2 binds to EP4 receptor on the sensory nerve, the signal is transduced to hypothalamus via DRG, resulting in CREB phosphorylation in the hypothalamus (13). The phosphorylated CREB (p-CREB) in hypothalamus can enhance peripheral bone formation by suppressing sympathetic nervous system activity. Here, *in vivo* and *in vitro* experiments were performed to determine the involvement of neurosensory PGE2/EP4/p-CREB axis in the JKSQP-induced OPF healing. IF analysis revealed significant upregulation of EP4 expression in both fracture callus and DRG, marked enhancement of CGRP level in the DRG and increased p-CREB in the hypothalamus after JKSQP treatment (**Figures 6A–F**). Furthermore, DRG neurons were isolated from 8-week-old female C57BL/6J mice, and subsequently treated with JKSQP-free or high JKSQP-containing serum treated BMSCs-conditioned medium, or the serum from OPF mice or JKSQP-treated mice (**Figure 6G**). DRG neurons were identified through morphological observation and IF staining. Under the microscope, DRG neurons exhibited characteristic oval cell bodies with radiating neurites (**Figure 6H**). β3-tubulin, a neuron-specific cytoskeletal marker, was largely expressed in DRG neurons, while few GFAP expression served to exclude potential astrocyte contamination (**Figure 6I**). The results of qRT-PCR and IF staining showed that the expressions of EP4 and CGRP were significantly increased in the DRG neurons after administration of high JKSQP-containing serum treated BMSCs-conditioned medium and the serum from JKSQP-treated mice (**Figures 6J, K**). All these findings indicate that JKSQP accelerates OPF healing in mice possibly through activation of neurosensory PGE2/EP4/p-CREB axis.

## 4 Discussion

With the growth of the aging population, OPFs have become a major public health challenge due to its high disability rate and the lack of effective therapeutic agents (25). Traditional Chinese medicine (TCM), primarily composed of natural products, is recognized as a valuable resource for developing novel drugs with improved efficacy and safety profiles (26). Guided by the “kidney governing bones” theory in TCM (16), JKSQP, a kidney-tonifying herbal formula, has demonstrated significant clinical efficacy in OPFs management. In this study, we systematically investigated the pharmacological mechanisms of JKSQP using integrated an OPF mouse model and *in vitro* cellular experiments. μCT analysis and ABH staining on fracture callus at three postoperative time points (days 7/14/21) demonstrated accelerated fracture healing in JKSQP-treated mice. Calcein double-labeling and upregulated Osterix expression confirmed enhanced bone formation in the fracture callus, which mechanistically accelerated mouse OPF healing. Elevated PGE2 levels both in the serum of JKSQP-treated mice and conditioned medium from BMSCs intervened with JKSQP-containing serum, enhanced EP4 expression in the DRG JKSQP-treated mice and primary DRG neurons, and high expression of p-CREB in hypothalamus, revealed that JKSQP promotes bone formation during OPF healing possibly through activating neurosensory PGE2/EP4/p-CREB axis.

OPFs must be established on a pathological foundation of osteoporosis. Bilateral OVX, the gold standard for inducing postmenopausal osteoporosis, reliably produces significant bone loss beginning at 4 weeks postoperatively (27). In our study, we validated substantial bone mass reduction in OVX mice 8 weeks post-surgery using µCT analysis. Subsequently, a standardized tibial osteotomy procedure was performed to successfully establish the OPF model. This mouse OPF model aligns with established methodologies (28–30), in which transverse mid-diaphyseal femoral/tibial fractures are induced following 4–12 weeks of osteoporotic induction.

OPF healing progresses through three overlapping phases including hematoma, callus formation and bone remodeling, mediated by two distinct processes including endochondral ossification and intramembranous ossification (31–33). To analyze the therapeutic mechanism of JKSQP, longitudinal analyses were conducted at 4, 14, and 24 days post-fracture that was corresponded to these defined healing phases as established in previous studies. At 4 days post-fracture, early cartilaginous callus formation was observed in JKSQP-treated mice compared to the OPF controls, indicating that callus initiation was advanced by JKSQP. At 14 days post-fracture, a larger cartilaginous callus was formed at the fracture gap, accompanied by a bigger woven bone deposition at the periphery of the callus after JKSQP treatment. These observations were consistent with the spatial heterogeneity in fracture healing: The relatively unstable fracture gap heals via endochondral ossification, where a cartilage template is initially formed and subsequently replaced by bone (8); The mechanically stable regions at the callus periphery heal through intramembranous ossification, directly forming woven bone (9). Enhanced bone mineralization (as demonstrated by Calcein double-labeling) combined with markedly upregulated Osterix expression, indicated accelerated osteogenesis in the fractured callus after JKSQP treatment. This enhancement of bone formation explains the complete fracture union observed in JKSQP-treated mice by 24 days post-fracture, whereas untreated controls exhibited persistent remodeling without full consolidation. Collectively, these findings demonstrate that JKSQP can promote bone formation to accelerate OPF healing. To our knowledge, no previous studies have investigated the effects of JKSQP on any bone disorders. Notably, JKSQP shares therapeutic characteristics with other documented kidney-tonifying herbs and formulas (e.g., Bushen Huoxue formula (34), Eucommia ulmoides (35), Epimedium (36)) that have demonstrated osteogenic activity in preclinical models. This observed bone-forming capacity of JKSQP aligns with the foundational TCM theory of “kidney governing bones”.

**Figure 7:**
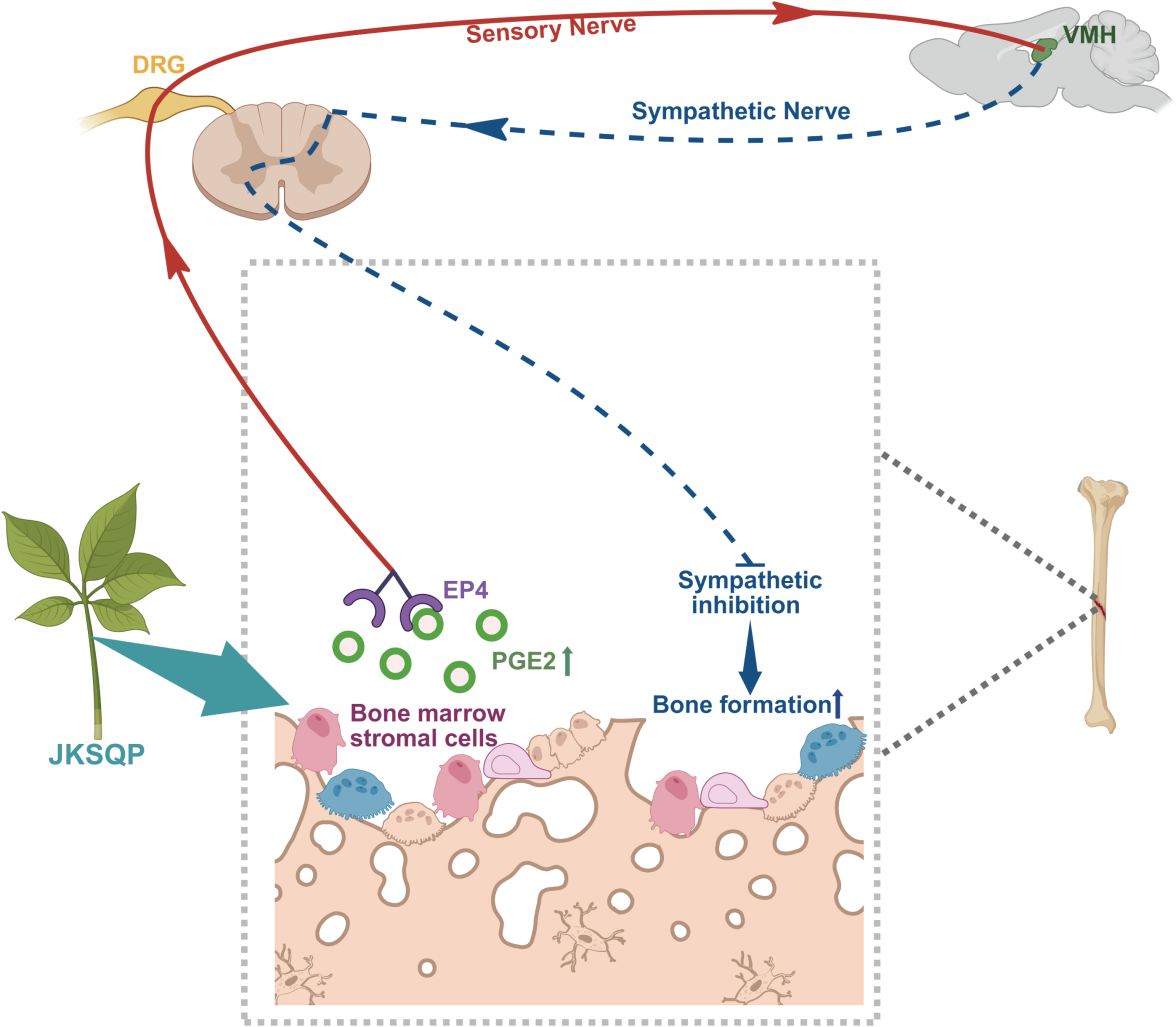
Mechanistic framework of JKSQP in osteoporotic fracture (OPF) healing. JKSQP enhances PGE2 synthesis by upregulating COX-2 expression in bone marrow stromal cells (BMSCs). The elevated PGE2 binds to prostaglandin E receptor 4 (EP4) on dorsal root ganglion (DRG) neurons, activating hypothalamic phosphorylated cAMP response element-binding protein (p-CREB). This signaling cascade suppresses central nervous system-mediated sympathetic nervous activity, thereby promoting osteogenic regeneration and accelerating OPF repair.

Sensory nerves play a dual regulatory role in skeletal physiology, extending beyond nociception to critically modulate fracture repair processes (11, 37). Emerging evidence demonstrates that sensory denervation disrupts osteogenesis, leading to structurally deficient callus formation with compromised biomechanical integrity (12). The neurosensory PGE2/EP4/p-CREB axis has been identified as critical for mediating sensory nerve-regulated bone formation. Specifically, PGE2 binds to EP4 receptor on sensory nerves, triggering signal transduction to the hypothalamus via DRG. This cascade activates CREB phosphorylation in the hypothalamus, which subsequently suppresses sympathetic nervous activity, thereby creating an osteogenic microenvironment within bone (13, 14). *In vivo* experiments demonstrated significant up-regulation of COX-2 within fracture callus tissue and elevated serum PGE2 levels in JKSQP-treated mice compared to OPF controls. BMSCs, critical contributors to fracture healing, were identified as the primary cellular source of PGE2 synthesis. *In vitro* experiments using primary BMSCs demonstrated that JKSQP enhances PGE2 synthesis through activation of COX-2 in BMSCs. Furthermore, JKSQP administration significantly increased EP4 receptor expression in primary DRG neurons and DRG tissues isolated from OPF mice, concomitant with enhanced p-CREB in the hypothalamus. Integratively, JKSQP accelerates OPF healing through a coordinated cascade (**Figure 7**): Upregulating COX-2 in BMSCs augments PGE2 synthesis; The elevated PGE2 binds to EP4 receptor on DRG neurons to activate hypothalamic p-CREB; This signaling cascade suppresses central nervous system-mediated sympathetic nervous activity, thereby promoting osteogenesis and accelerating OPF repair.

Nevertheless, there were some limitations in this study. Firstly, the pharmacological evaluation employed a single dosage based on established human-mouse dose conversion guidelines, potentially overlooking dose-dependent effects. Secondly, although LC-MS/MS analysis identified 1872 chemical components in JKSQP aqueous extract, we did not characterize pharmacologically active metabolites in serum/urine post-administration. Thirdly, this study focused primarily on morphological assessment of OPF healing via micro-CT and histology, missing the functional or pain-related evaluations, such as three-point bending mechanical testing, gait analysis and open field test. Lastly, the therapeutic specificity of the PGE2/EP4/p-CREB axis would be strengthened by incorporating *in vivo* rescue studies using pathway-specific inhibitors to confirm mechanism-dependent efficacy.

In summary, this study demonstrates that JKSQP accelerates OPF healing by enhancing bone formation through activation of the neurosensory PGE2/EP4/p-CREB signaling axis.

## Data Availability

The raw data supporting the conclusions of this article will be made available by the authors, without undue reservation.

## Supplementary material

Supplementary Figure 1 μCT images of fracture callus formation in the JKSQP treated mice at 4, 14 and 24 days post-fracture.
