# Supplementary figures and images for: Jinkui Shenqi Pill accelerates osteoporotic fracture healing by promoting bone formation through neurosensory PGE2/EP4/p-CREB axis
Source: Front Endocrinol (Lausanne). 2025 Jul 4;16:1570685. doi: 10.3389/fendo.2025.1570685 (PMC12270888; doi:10.3389/fendo.2025.1570685)

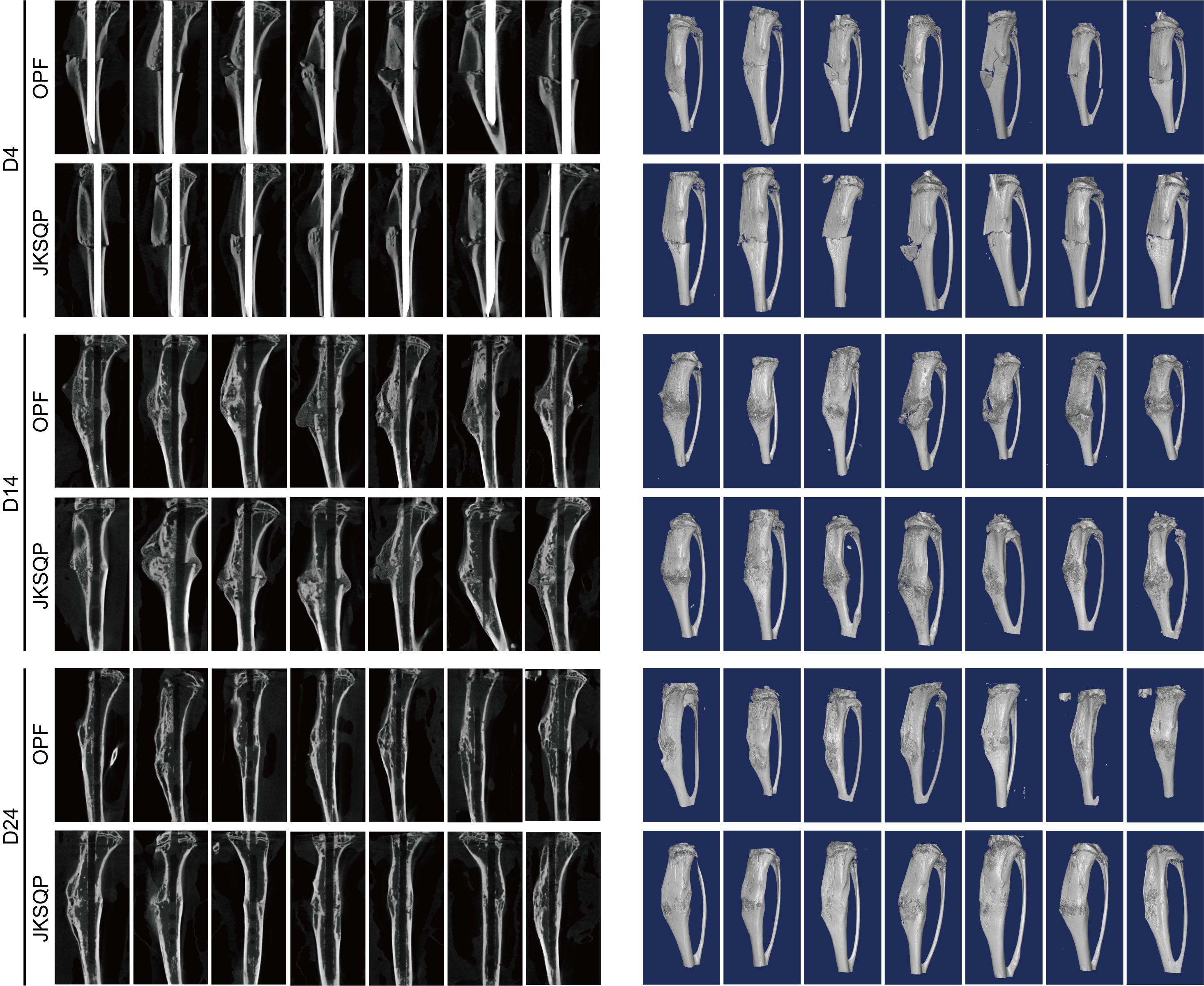

Supplement: Supplementary Figure 1 — μCT images of fracture callus formation in the JKSQP treated mice at 4, 14 and 24 days post-fracture. [file Image1.tif]
